# Post-Ugi Cyclization for the Construction of Diverse Heterocyclic Compounds: Recent Updates

**DOI:** 10.3389/fchem.2018.00557

**Published:** 2018-11-20

**Authors:** Jitender Bariwal, Rupinder Kaur, Leonid G. Voskressensky, Erik V. Van der Eycken

**Affiliations:** ^1^Shiva Institute of B. Pharmacy, Bilaspur, India; ^2^Department of Pharmaceutical Chemistry, ISF College of Pharmacy, Moga, India; ^3^Laboratory for Organic & Microwave-Assisted Chemistry, University of Leuven, Leuven, Belgium; ^4^Peoples' Friendship University of Russia (RUDN University), Moscow, Russia

**Keywords:** Post-Ugi modifications, heterocycles, multicomponent reactions, MCR, post-Ugi cyclization

## Abstract

Multicomponent reactions (MCRs) have proved as a valuable tool for organic and medicinal chemist because of their ability to introduce a large degree of chemical diversity in the product in a single step and with high atom economy. One of the dominant MCRs is the Ugi reaction, which involves the condensation of an aldehyde (or ketone), an amine, an isonitrile, and a carboxylic acid to afford an α-acylamino carboxamide adduct. The desired Ugi-adducts may be constructed by careful selection of the building blocks, opening the door for desired post-Ugi modifications. In recent times, the post-Ugi transformation has proved an important synthetic protocol to provide a variety of heterocyclic compounds with diverse biological properties. In this review, we have discussed the significant advancements reported in the recent literature with the emphasis to highlight the concepts and synthetic applications of the derived products along with critical mechanistic aspects.

## Introduction

Multicomponent reactions (MCRs) are considered as privileged one-pot processes involving a sequential combination of at least three reagents in the same pot (Ramon and Yus, 2005). The MCRs have found their usefulness and influence in Diversity-Oriented Synthesis (DOS) particularly in the field of medicinal chemistry (Biggs-Houck et al., 2010). The combinatorial compound libraries play a vital role for achieving the goal of DOS providing large compound libraries with reduced synthetic steps and with increased molecular complexity (Bariwal et al., 2010).

**Scheme 1:**
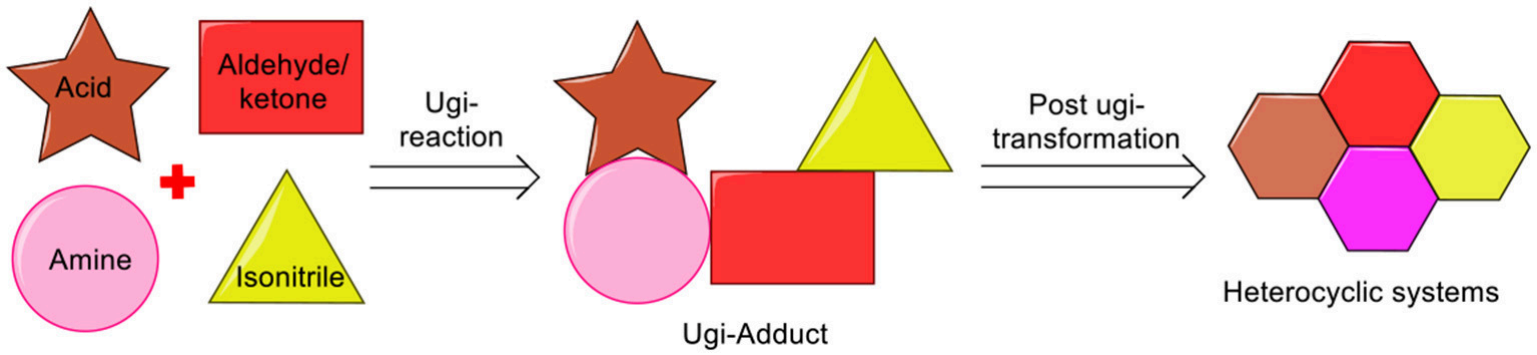
Ugi multicomponent reaction and post Ugi transformation to heterocyclic system.

From the past two decades, the Ugi four-component reaction (Ugi-4CR) has offered one of the most investigated reaction route for generating multifunctional adducts, owing to the mildness of the reaction conditions, the wide application and the high variability (four diversity points) associated with it (Ugi and Steinbrückner, 1961; Ugi, 1962). More importantly, the Ugi-adduct can be manipulated by careful selection of the starting components (amine, acid, isonitrile, and aldehyde/ketone). Synthesis of the desired heterocycle can be achieved by performing a post-Ugi transformation on the exclusively designed Ugi-adduct (Sharma et al., 2015). The efficiency of post-Ugi transformation is further enhanced by manipulating the Ugi-adducts in a regioselective manner to provide access to complex heterocycles in a domino fashion (Li et al., 2016) (Scheme 1).

There are several reports for the transformations of the Ugi-adducts to diverse heterocyclic systems including Ugi-Heck, Ugi-ring opening metathesis, Ugi-intramolecular arylation, post-Ugi-cascades, and metal/ligand regio-, chemoselectivity switch, which has been reviewed by us recently (Sharma et al., 2015) and others (Hulme and Dietrich, 2009; Kaïm and Grimaud, 2010, 2014; Koopmanschap et al., 2014; Xiuming et al., 2017). During the last few years, noteworthy progress in the post-Ugi-transformations has taken place as is evident from the substantial number of reports. We have carefully selected the prime literature reports where Ugi-adducts have been cyclized to generate a variety of heterocyclic systems. The present review mainly covers the literature reports on post-Ugi-modifications appeared after the year 2014. However, we have included few other important reports before the year 2014 and were not discussed in previous reviews. We have grouped these reports based on the structural complexity of the product after cyclization. For more clarity, we have started the discussion with the construction of the small heterocyclic systems to larger, and then followed by fused systems after post-Ugi-cyclization as given below:

1. Four-membered heterocycles
2. Five-membered heterocycles
3. Six-membered heterocycles
4. Fused heterocycles
  4.1 Five-membered heterocycles fused with five-membered heterocyclic systems
  4.2 Five-membered heterocycles fused with six-membered carbocyclic systems
  4.3 Five-membered heterocycles fused with six-membered heterocyclic systems
  4.4 Five-membered heterocycles fused with medium sized heterocyclic systems
  4.5 Six-membered heterocycles fused with six-membered carbocyclic systems
  4.6 Seven-membered heterocycles fused with six-membered carbocyclic systems
  4.7 Nine-membered heterocycles fused with five-membered heterocycles
5. Tricyclic fused heterocycles
6. Spiro-polyheterocycles

## Four-membered heterocycles

**Scheme 2:**
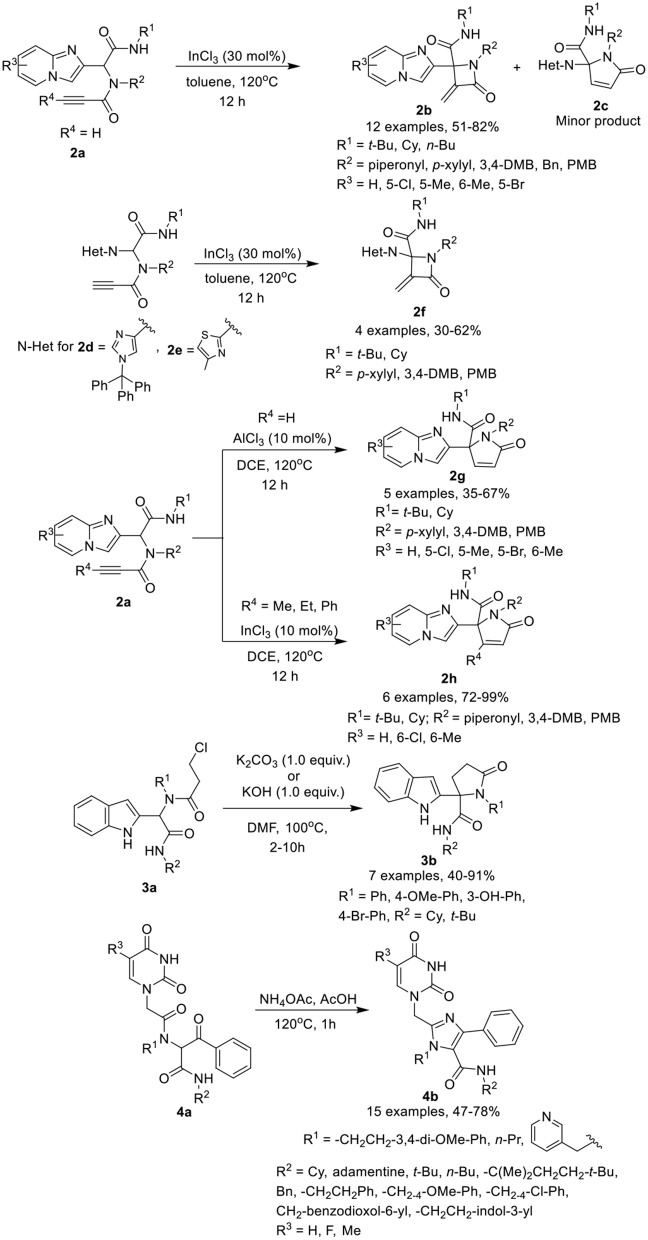
Synthesis of fused α-methylene β-lactams **2b, 2f**, α,β-unsaturated γ-lactams **2g, 2h**, indolyl based γ-lactams **3b** and tetra-substituted Imidazoles **4b**.

2-Azetidinones, known as β-lactams, are well known antimicrobial agents and are important synthetic intermediates for vitamins, alkaloids, and β-amino acids. Van der Eycken et al. (Li et al., 2015b) have developed a diversity-oriented post-Ugi intramolecular cyclization of Ugi-adducts **2a** to give access to α-methylene β-lactams **2b** in moderate to excellent yields using InCl_3_ as a catalyst in toluene at 120°C with α,β-unsaturated γ-lactams **2c** as a minor product (Scheme 2). Under the optimized conditions, the intramolecular cyclization reaction proceeded smoothly to give α-methylene β-lactams utilizing a variety of substituents on the Ugi-adducts. Ugi-adducts (**2d** and **2e**) prepared from 1-trityl-1*H*-imidazole-4-carbaldehyde and 4-methylthiazole-2-carbaldehyde provided the corresponding alkylidene-β-lactams **2f** in good yields. However, Ugi-adduct derived from imidazo[1,2-*a*]pyridine-3-carbaldehyde or from benzaldehyde failed to cyclized under these conditions. Moreover, the use of Ugi-adducts derived from 4-pentynoic acid did not provide the desired alkylidene-β-lactam.

Interestingly, switching the catalyst to AlCl_3_, provided exclusive access to the unsaturated γ-lactams **2g** in moderate to excellent yields. Under the optimized conditions, the Ugi-adducts obtained from substituted imidazo[1,2-*a*]pyridine-2-carbaldehydes provided moderate to good yields, whereas 1-trityl-1*H*-imidazole-4-carbaldehyde derived Ugi-adduct provided a low product yield (20% only). In addition, Ugi-adducts **2a** prepared from imidazo[1,2-*a*]pyridine-2-carbaldehyde and 2-butynoic acid, underwent the InCl_3_-catalyzed Michael addition reaction (InCl_3_ (10 mol%) in DCE at 120°C for 12 h) and provided exclusive access to γ-lactam **2h** in good to excellent yields, even with a bulky substituent such as a phenyl group on the alkyne.

## Five-membered heterocycles

Shiri et al. (2014) have reported a selective synthesis for a series of novel indolyl based γ-lactams **3b** by cyclization of Ugi-adducts **3a** in the presence of K_2_CO_3_ in DMF at 100°C within few hours (Scheme 2). Under the optimum conditions, adducts derived from activated anilines underwent the reaction smoothly and gave access to the corresponding lactams in moderate to good yields. However, synthesis of Ugi-adducts using deactivated anilines met with failure. Benzyl aniline derived Ugi-adduct also afforded the desired *N*-benzyl lactam in excellent yield, whereas benzaldehyde-derived Ugi-adduct required modified conditions (KOH (1.0 equiv) DMF, 100°C, 10h) to provide access to the γ-lactam in moderate yield. Bulky t*ert*-butyl isocyanide-derived Ugi-adduct significantly reduced the product yield compared to the cyclohexyl substituted substrate.

Dömling et al. (Madhavachary et al., 2018) have developed a new, facile one-pot synthetic approach for the synthesis of uracil/thymine-containing tetra-substituted imidazoles **4b** by cyclizing Ugi-adducts **4a** with NH_4_OAc in AcOH at 120°C in moderate to excellent yields (Scheme 2). Under the optimized conditions, a variety of substituted isocyanides, aliphatic, and aromatic amines and uracil derived acetic acids were found suitable for the reaction and provided good to excellent product yields. From this small library, products obtained from 5-fluorouracil and 5-methyluracil acetic acids are interesting analogs of the marketed drugs Retrovir and Tegafur and were obtained in only two synthetic steps.

## Six-membered heterocycles

**Scheme 3:**
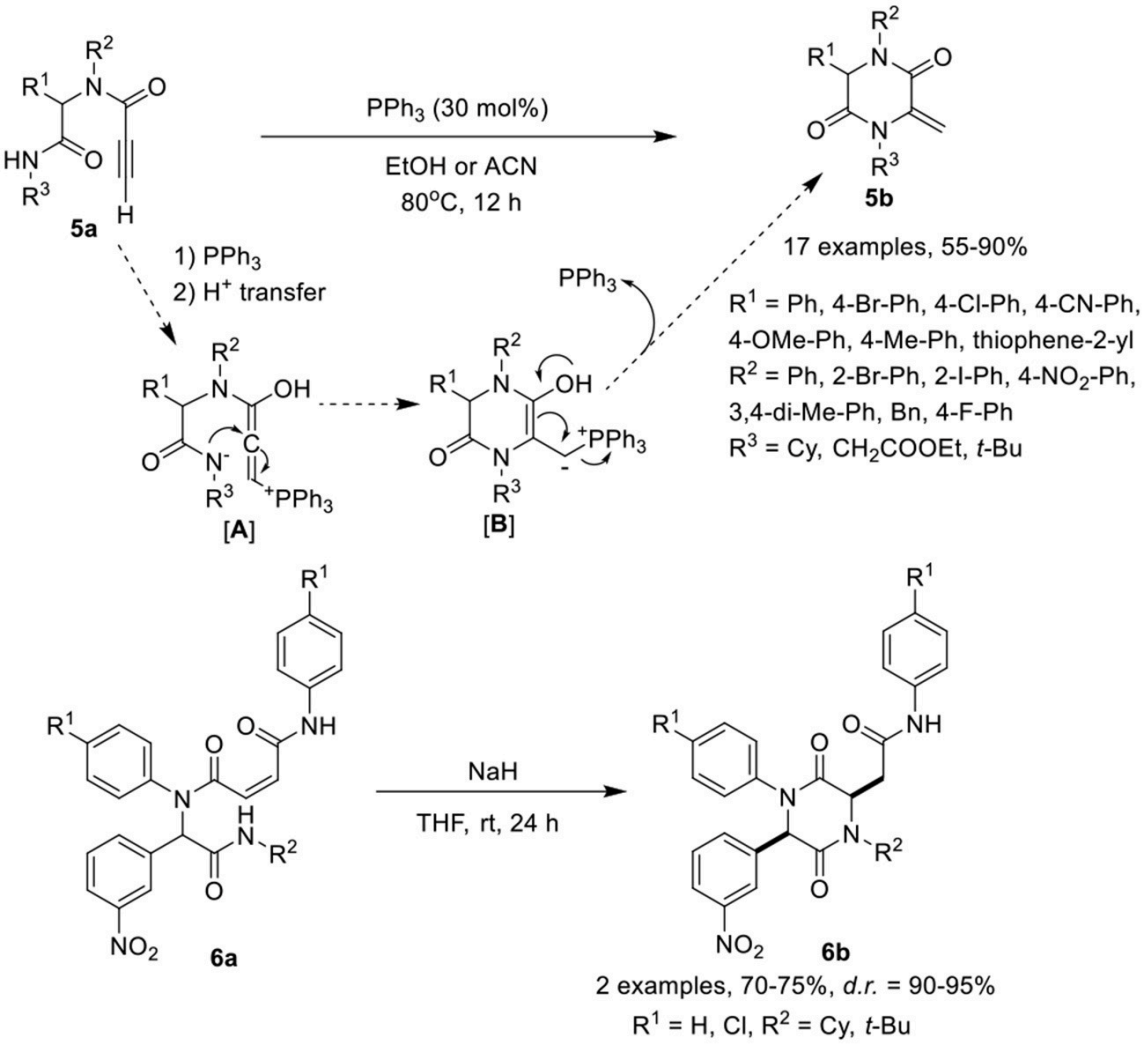
Synthesis of arylidence 2,5-diketopiperazines **5b** and piperazine 2,5-dione **6b**.

Balalaie et al. (2017a) have developed a facile post-Ugi-transformation to cyclize Ugi-adducts **5a** to 2,5-diketopiperazines **5b** in good to excellent yields in the presence of triphenylphosphine as catalyst in ethanol at 80°C (Scheme 3). Under the optimized conditions, various Ugi-adducts derived from aldehydes and anilines bearing electron-withdrawing and electron-releasing substituents performed well to give the desired products in good to high yields, except with thiophene-2-carbaldehyde and 2-iodo substituted aniline which were obtained in moderate yields. Adducts derived from isocyanides bearing cyclohexyl or ethyl ester underwent the reaction smoothly. However, with *tert*-butyl bearing isocyanide, acetonitrile as solvent was required to furnish the corresponding 2,5-diketopiperazines in moderate yields. The post-Ugi cyclization reaction proceeds by nucleophilic addition of triphenylphosphine to the active alkyne group followed by the proton transfer from amide NH as shown in [**A**]. The nucleophilic addition of nitrogen results in the formation of cyclized intermediate [**B**], followed by PPh_3_ elimination furnishing the desired 2,5-diketopiperazines **5b**.

Halimehjani and Sharifi (2017) have reported a simple approach to synthesize functionalized piperazine 2,5-diones **6b** via intramolecular aza-Michael addition reaction of Ugi-adducts **6a** in the presence of NaH in THF at rt (Scheme 3). Under the optimized conditions, the reaction proceeds smoothly and provides high product yields with excellent diastereoselectivity.

## Fused heterocycles

### Five-membered heterocycles fused with five-membered heterocyclic systems

**Scheme 4:**
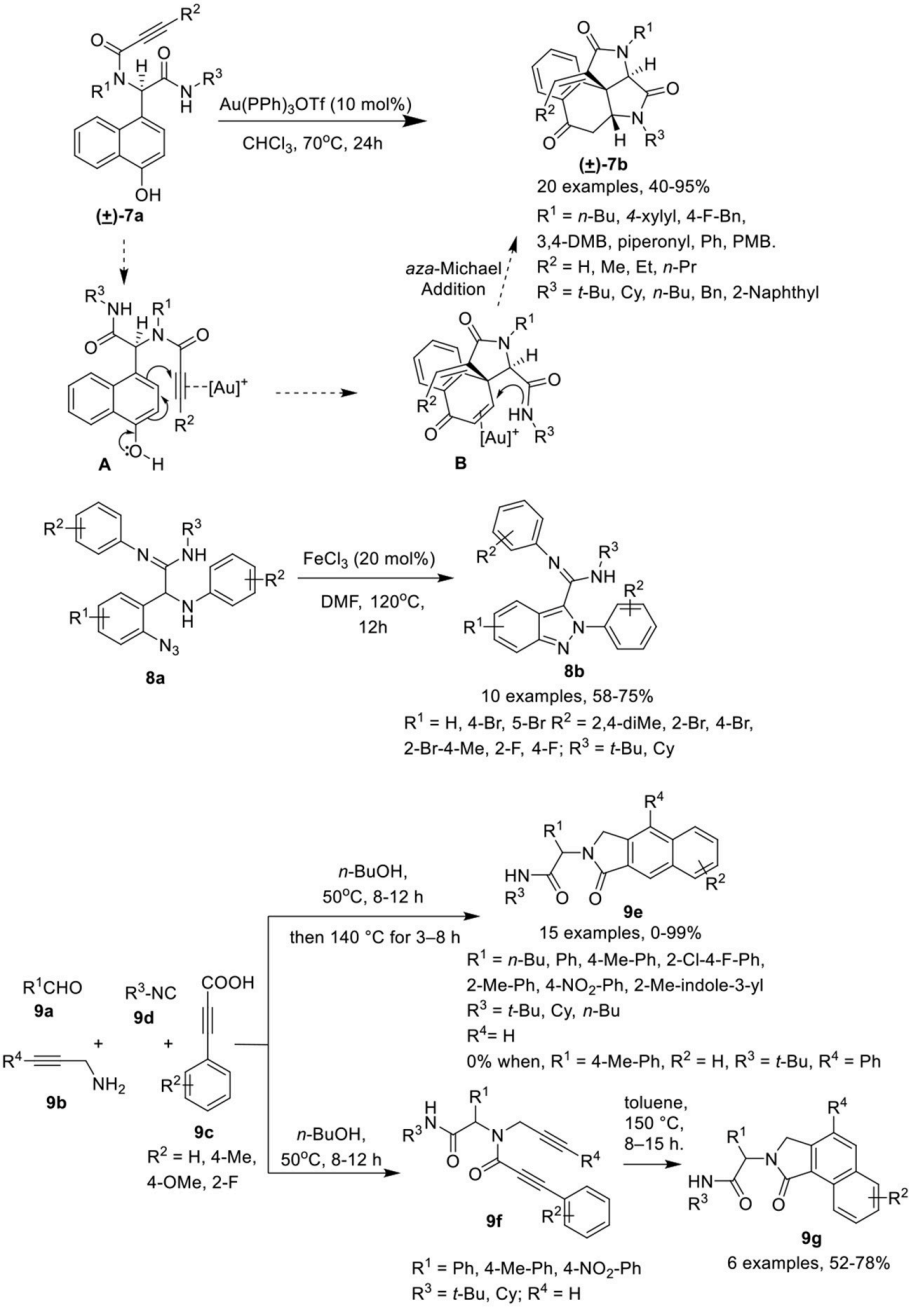
Synthesis of azaspiro tetracyclic scaffolds **7b**, amidino substituted indazoles **8b** and *N*-substitutednenzo*[e]-*or *[f]*isoindolones **9e,g**.

Li et al. (He et al., 2017) have developed a facile post-Ugi gold(I)-catalyzed domino dearomatization/*ipso*-cyclization/aza-Michael cascade reaction of diverse Ugi-adducts **7a** to gain access to the functionalized tetracyclic benzo[*e*]pyrrolo[2,3-*c*]indole-2,4,7(5*H*)-triones **7b** in good yields and with unique diastereoselectivities. The reaction gave best results when Au(PPh_3_)OTf was used as a catalyst in CHCl_3_ at 70°C (Scheme 4). Under the optimized conditions, a variety of Ugi-adducts **7a** prepared from diversely substituted amines, isocyanides and acids bearing electron-withdrawing or electron-donating groups performed well to give the desired products in good to excellent yields. Interestingly, Ugi-adducts derived from isonitriles bearing a bulky substituent did not significantly affected the reaction yield. The substrates comprising a terminal alkyne also gave the *exo*-dig products in moderate to good yields. The gold(I)-catalyzed domino dearomatization/*ipso*-cyclization/aza-Michael sequence proceeded by *in situ* formation of a cationic gold(I) species which is followed by nucleophilic attack by the C-4 position of the 1-naphthol (5-*exo*-dig fashion) as shown in intermediate [**A**]. This leads to the formation of the spirocarbocyclic intermediate [**B]**. This generated the tetracyclic scaffold *via* aza-Michael addition facilitated by π-activation by the cationic gold species.

### Five-membered heterocycles fused with six-membered carbocyclic systems

Sharada et al. (Sagar et al., 2015) have described a FeCl_3_ catalyzed post-Ugi cyclization protocol for cyclization of α-amino amidines **8a**, generated *via* an Ugi-3CR using silica gel as promoter, to construct amidino substituted indazoles **8b** in good to high yields in DMF at 120°C (Scheme 4). Under the optimized conditions, a variety of amidino substituted indazoles were prepared where the electronic nature of substituents on the various Ugi-components did not hamper the reaction and provided good product yields.

Van der Eycken et al. (Ambasana et al., 2014) have reported a solvent switchable metal-free [4 + 2] cycloaddition reaction *via* ${\text{C}}_{\text{sp}}^{2}$-H functionalization of the Ugi-adduct, *N*-propynylphenyl propiolamide **9f** (prepared by reacting aldehyde **9a**, amine **9b**, 2-alkynoic acid **9c** and isonitrile **9d**), to access the *N*-substituted benzo[*f* ]isoindolone **9e** or benzo[*e*]isoindolone **9g** in excellent yields and with good regioselectivity. The cyclization reaction to access the benzo[*e*]isoindolones **9g** gave best results in toluene at 150°C. However, the use of a polar-protic solvent alone, or with toluene as co-solvent, resulted in an increased formation of benzo[*f* ]isoindolone **9e**. Interestingly, use of *n*-BuOH as a solvent in a one-pot reaction for Ugi-4CR and ring closure reaction, provided access to selective formation of **9e** over **9g** (Scheme 4). Under the optimized conditions, Ugi-adducts generated from a broad range of aromatic aldehydes, isonitriles and acids performed well to furnish the benzo[*f* ]isoindolones **9e** in excellent yields. However, a low yield of **9e** was observed when aliphatic aldehyde- such as valeraldehyde- derived Ugi-adducts were used in the reaction, whereas use of Ugi-adduct derived from phenyl propargylamine did not underwent the reaction. Further, variedly substituted benzo[*e*]isoindolones **9g** were prepared in moderate to high yields under the optimized conditions from Ugi-adducts derived from differently substituted aldehydes, acids and isonitriles. Importantly, an electron-withdrawing group on the acids improves the yields, while the use of an electron deficient aldehyde and electron rich acid provides low product yields. Interestingly, employing *o*-substituted acid derived Ugi-adduct under the optimized conditions led to the formation of product **9g** as a single isomer.

### Five-membered heterocycles fused with six-membered heterocyclic systems

**Scheme 5:**
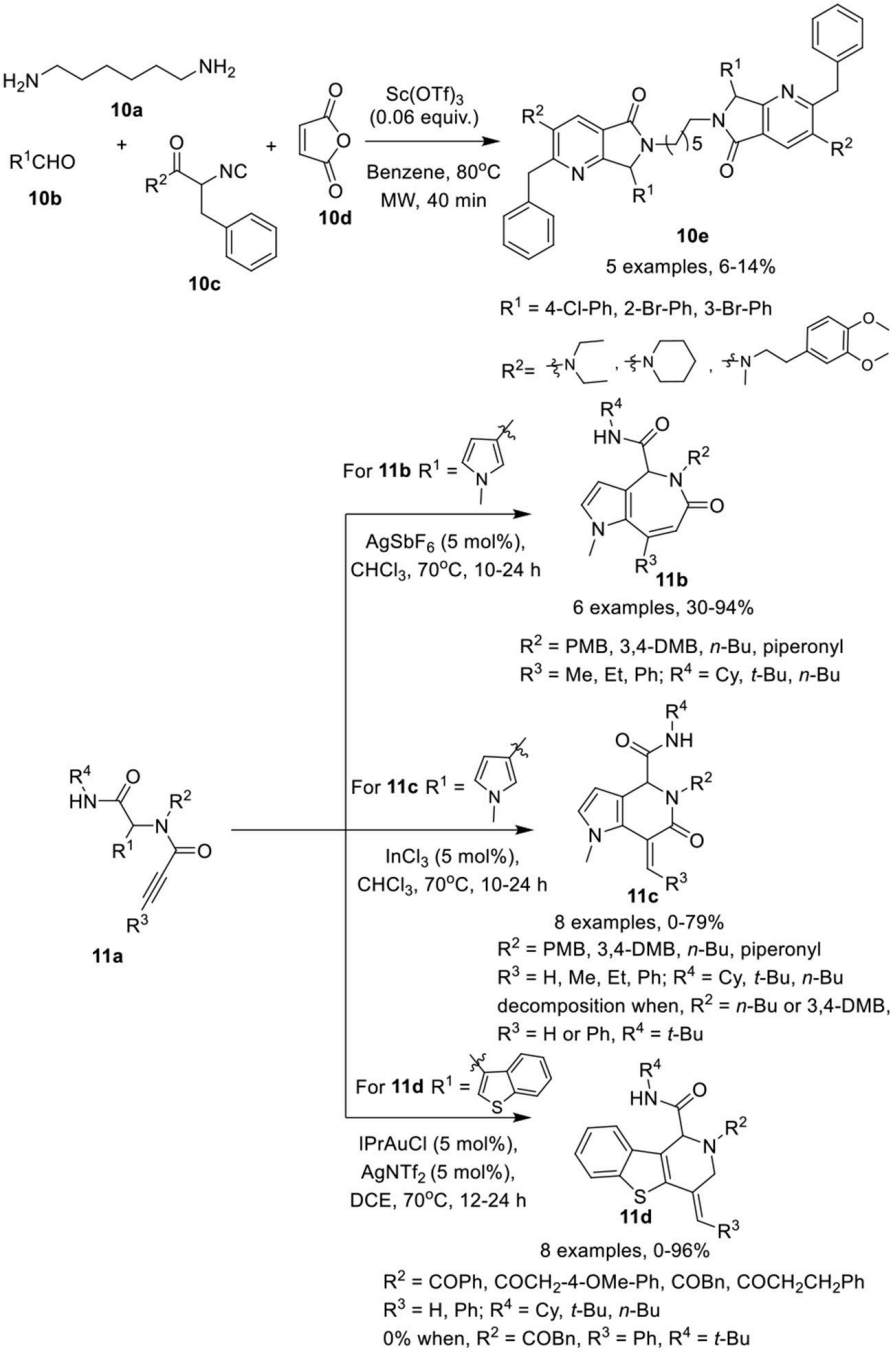
Synthesis of cyclic analogs of HMBPA 10e, pyrroloazepinones **11b**, pyrroloazepinones **11c**, and benzothienopyridines **11d**.

González-Zamora et al. (Zamudio-Medina et al., 2015) have described a one-pot, Ugi-4CR for the synthesis of cyclic analogs of hexamethylenebis(3-pyridine)amide (HMBPA) **10e** in low yields. After reaction of diamine **10a**, aldehydes **10b**, and isocyanoacetamides **10c**, cycloaddition and ring opening reaction with maleic anhydride **10d** was performed under MW irradiation using Sc(OTf)_3_ as a catalyst in benzene (Scheme 5). Under the optimized conditions, different isocyanoacetamides and aldehydes furnished the corresponding products **10e** in low yields. During this one-pot synthetic operation, six new chemical bonds were formed.

Van der Eycken et al. (Li et al., 2015a) have developed a facile catalyst-controlled regioselective process for post-Ugi intramolecular hydroarylation reaction to provide access to heterocycles such as pyrroloazepinones **11b**, pyrrolopyridinones **11c**, and benzothienopyridines **11d** in high yields. The intramolecular hydroarylation of Ugi-adduct **11a** furnished the desired pyrroloazepinone **11b** and pyrrolopyridinone **11c** in a nearly 1:1 ratio when Au(PPh_3_)Cl and AgOTf were used as a catalytic system at 50°C in CDCl_3_. Switching the catalyst to AgSbF_6_ in CDCl_3_ at 70°C provided exclusive access to pyrroloazepinones **11b** in excellent yields (Scheme 5). Other silver and gold salts such as AgOTf, AgBF_4_, AgNTf_2_, AuCl_3_, or AuCl also provided product in moderate to good yield. Interestingly, the use of InCl_3_ as catalyst in CDCl_3_, resulted in a switch of selectivity and afforded the pyrrolopyridinones **11c** in excellent yields. Ugi-adducts prepared from aliphatic *n*-butylamine or bulky alkyne substituents, such as phenyl, were not found suitable under these conditions resulting in unstable pyrrolopyridinones. On the other hand, for the pyrroloazepinone **11b**, diversely substituted Ugi-adducts performed well and provided good product yields. However, a bulky phenyl substituent on the alkyne, resulted in a drastic reduction of the product yield, whereas a terminal alkyne afforded the corresponding product in moderate yields. An electron-donating substituent on the carboxamide moiety provided excellent product yields in both cases. In addition, the *exo*-dig cyclization of the benzothiophene comprised Ugi-adducts was also performed in the presence of IPrAuNTf_2_ as catalyst in DCE at 70°C, affording the selective benzothienopyridines **11d** in excellent yields, whereas Au(PPh_3_)OTf provided the products in moderate yield and InCl_3_, AgSbF_6_, or AuCl_3_ did not work for this conversion. Under the optimized conditions, a variety of Ugi-adducts obtained from acids and isonitriles were well tolerated. However, a bulky phenyl substituent on the alkyne failed to give the corresponding product, whereas electron-donating substituents on the amines proved beneficial in this reaction.

**Scheme 6:**
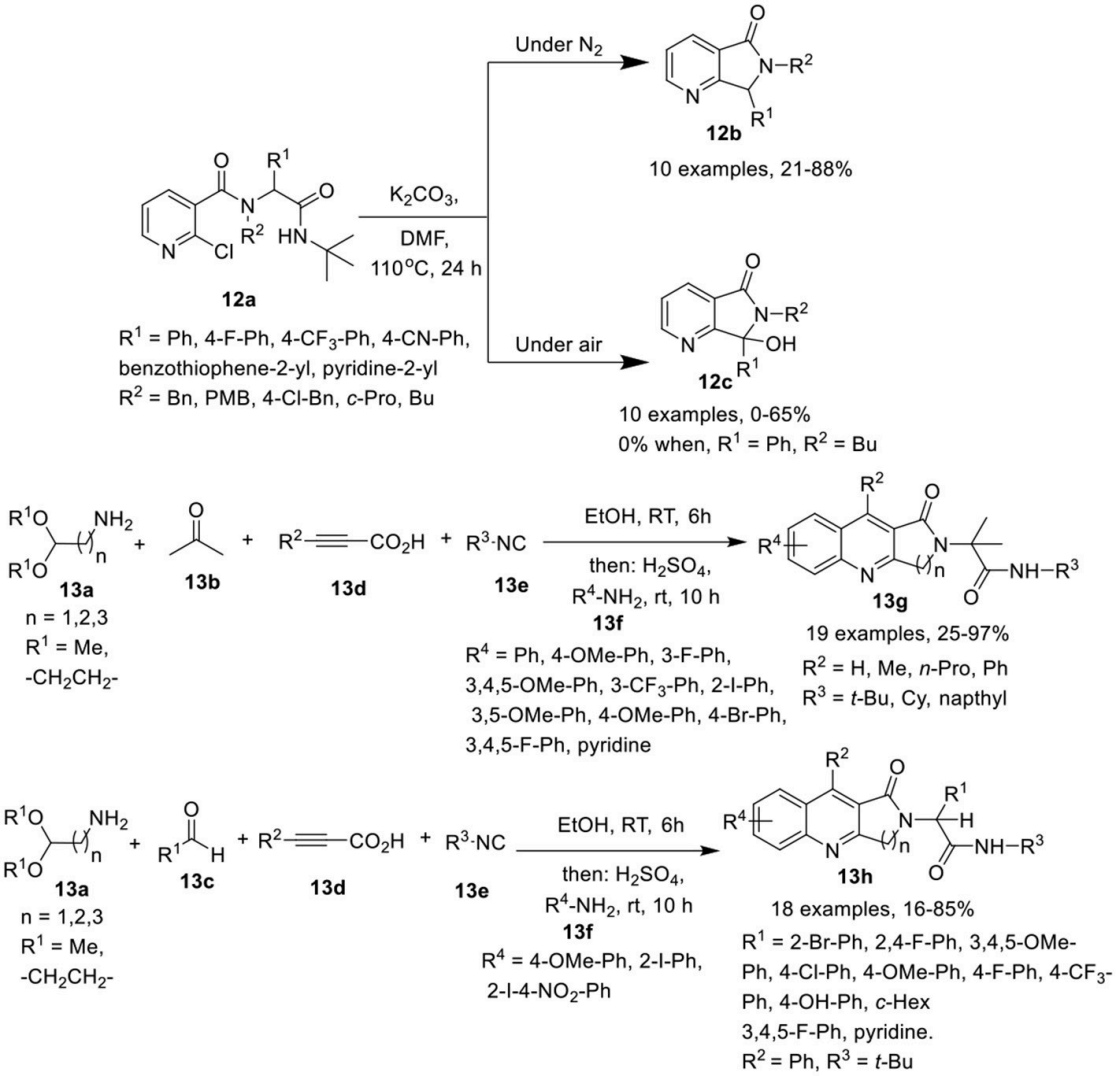
Synthesis of 6,7-dihydro-5*H*-pyrrolo[3,4-*b*]pyridine-5-ones **12b** and the oxidized product **12c** and fused quinolones **13g** and **13h**.

Peshkov et al. (Trang et al., 2015) have developed a one-pot base-promoted post-Ugi carbocyclization of Ugi-adduct **12a** with cleavage of the isocyanide-originated amide moiety, to provide facile access to 6,7-dihydro-5*H*-pyrrolo[3,4-*b*]pyridin-5-ones **12b** in high yields when performed under inert atmosphere at 110°C in DMF. However, when this reaction was performed under air atmosphere, the oxidized 7-hydroxy-6,7-dihydro-5*H*-pyrrolo[3,4-*b*]pyridin-5-one **12c** was obtained (Scheme 6). Adducts derived from a diverse array of aromatic amines as well as several aromatic and heteroaromatic aldehydes were found suitable in both inert and aerobic conditions, provided the desired products in good to moderate yields. However, aliphatic amines drastically reduced the product yield for both normal **12b** and oxidized product **12c**. Interestingly, the use of butylamine resulted in a poor yield of normal product **12b** and failed to give the oxidized product **12c**. In general, the yields for the non-oxidized products **12b** were higher than for the oxidized ones **12c**.

Srivastava et al. (Ghoshal et al., 2017) have reported an efficient and divergent one-pot sequential Ugi-4CR of amino acetaldehyde acetals **13a**, ketone **13b** or aldehydes **13c**, alkynoic acids **13d**, and isocyanides **13e** in ethanol and subsequent acid-mediated Povarov-type reaction followed by treatment with substituted anilines **13f** and H_2_SO_4_ to afford the fused quinolines **13g**, and **13h** in moderate to excellent yields at rt (Scheme 6). Under the optimized conditions, a diverse array of anilines bearing electron-rich as well as electron-deficient substituents were well tolerated in the Povarov-type reaction. However, heteroaromatic amines afforded the corresponding product in low yield. Interestingly, electron-deficient anilines such as 2-iodo-4-nitroaniline provided dihydro-2,3-dihydro-1*H*-pyrrolo[3,4-*b*]quinolin-1-ones (DHPQ) as a minor product which after oxidation, afforded the desired product in low yield. Among the isocyanides, *tert*-butylisocyanide and cyclohexylisocyanide were suitable, whereas the use of naphthylisocyanide led to the formation of the desired product in poor yield. For the acid counterpart, phenylpropiolic acid and other alkynoic acids were found suitable in the reaction resulting in good product yields. A diverse array of aromatic aldehydes bearing electron-rich or electron-deficient groups, as well as, aliphatic aldehydes were found suitable in this reaction, affording the corresponding products in moderate to excellent yields. Importantly, the acidic proton in the Ugi-adduct did not interfere in the Povarov-type reaction. Under these conditions, unprotected propiolamides also provided access to the corresponding products *via* Povarov-type reaction in moderate yields.

**Scheme 7:**
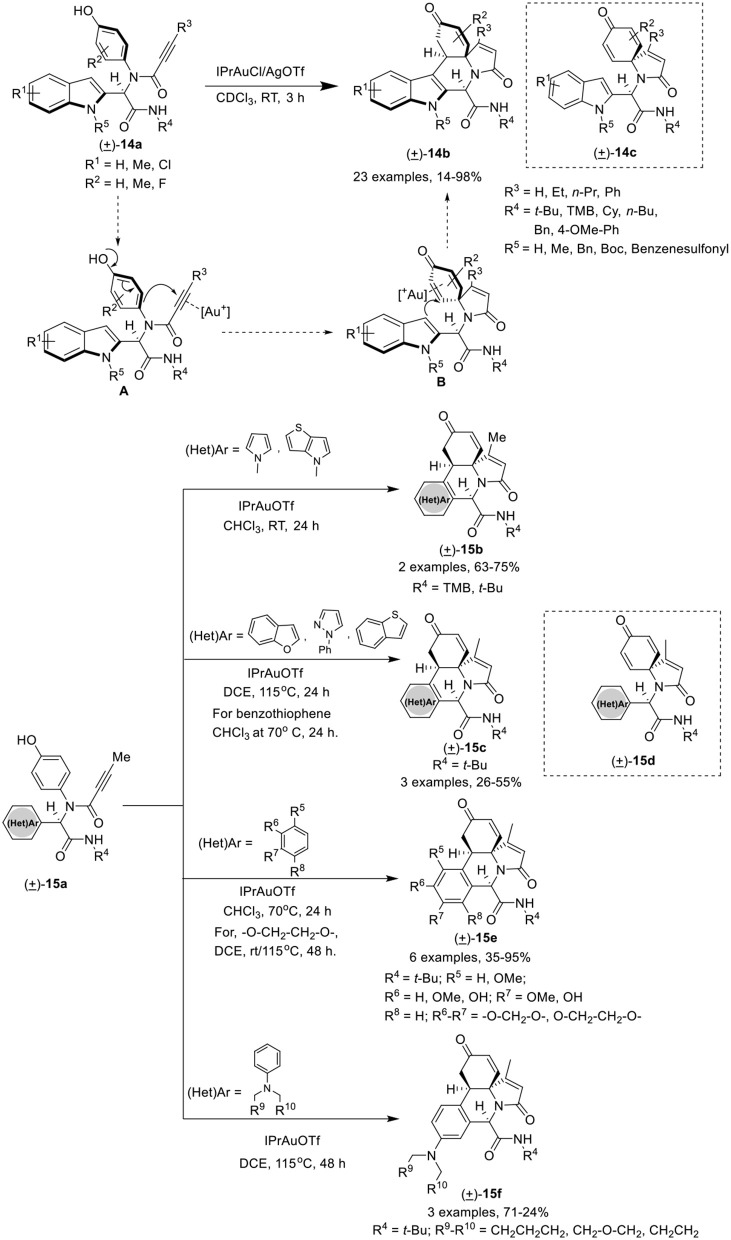
Synthesis of indole-annulated tricyclic heterocycle **14b** and diverse (hetero)-arene-annulated tricyclic heterocycle **15b-f**.

Van der Eycken et al. (He et al., 2018) have developed an efficient gold(I)-catalyzed post-Ugi domino dearo matization/*ipso*-cyclization/Michael sequence of Ugi-adduct **14a** that provided access to various (hetero)-arene-annulated tricyclic heterocycles **14b** in moderate to good yields with excellent chemo-, regio-, and diastereoselectivity. The reaction for the synthesis of indole-annulated tricyclic heterocycle **14b** proceeded smoothly utilizing IPrAuCl and AgOTf as catalytic system in CDCl_3_ at rt (Scheme 7). Under the optimal conditions, Ugi-adducts derived from aliphatic and aromatic isonitriles underwent the reaction smoothly and provided good product yields. Bulky alkyne substrates provided good product yields, whereas Ugi-adducts bearing a terminal alkyne did not performed well. Electron-donating as well as electron-withdrawing substituents on the indole ring were well tolerated and provided high yields. A strong electron-withdrawing benzenesulfonyl group on the indole nitrogen atom drastically reduced the C3 nucleophilicity, resulting in the need of high temperature (115°C) to perform the reaction in excellent yield. In contrast to Ugi-adducts derived from *ortho*-substituted 4-aminophenols, *meta*-substituted 4-aminophenols afforded low yields. Pyrrole-containing Ugi-adducts **15a** gave access to the corresponding pyrrole-fused polyheterocycles **15b** in good yields under the optimized conditions. In addition, different Ugi-adducts derived from various heteroaromatic aldehydes led to diverse (hetero)-arene-annulated tricyclic heterocycles in moderate to good yields using the modified conditions. The use of Ugi-adducts derived from benzofuran-2-carbaldehyde, 1-phenyl-1*H*-pyrazole-5-carboxaldehyde, and benzo[*b*]thiophene-3-carbaldehyde resulted in the formation of a mixture of heteroarene-annulated tricycle **15c** and spirocarbocyclic product **15d** in moderate to good yields upon heating at 115°C. Furthermore, Ugi-adducts derived from electron-rich aromatic aldehydes (hydroxy- and alkoxy-substituted benzaldehydes) when used in this domino process underwent the reaction smoothly in CHCl_3_ at 70°C to afford the benzene-annulated tricyclic heterocycles **15e** in moderate to good yields. The pyrrolidine-substituted benzene provided the benzene-annulated tricycle in high yield at rt, whereas, piperidine- or morpholine-substituted benzaldehydes afforded the benzene-annulated tricycles **15f** in DCE at 115°C in low yields. Interestingly, when these reactions were performed at rt, only the spirocarbocyclic products were obtained (Scheme 7). The reaction proceeds by *in situ* generation of cationic gold(I) species that π-activates the alkyne group in the Ugi-adducts to give intermediate [**A**], which is subsequently attacked by the nucleophilic phenol in a 5-*endo*-dig fashion to afford spirocarbocyclic intermediate [**B]**. Subsequent Michael addition of the intermediate cyclohexadienone with the C3 position of the indole, results in the formation of the indole-annulated tricyclic heterocycle **14b** (Scheme 7).

**Scheme 8:**
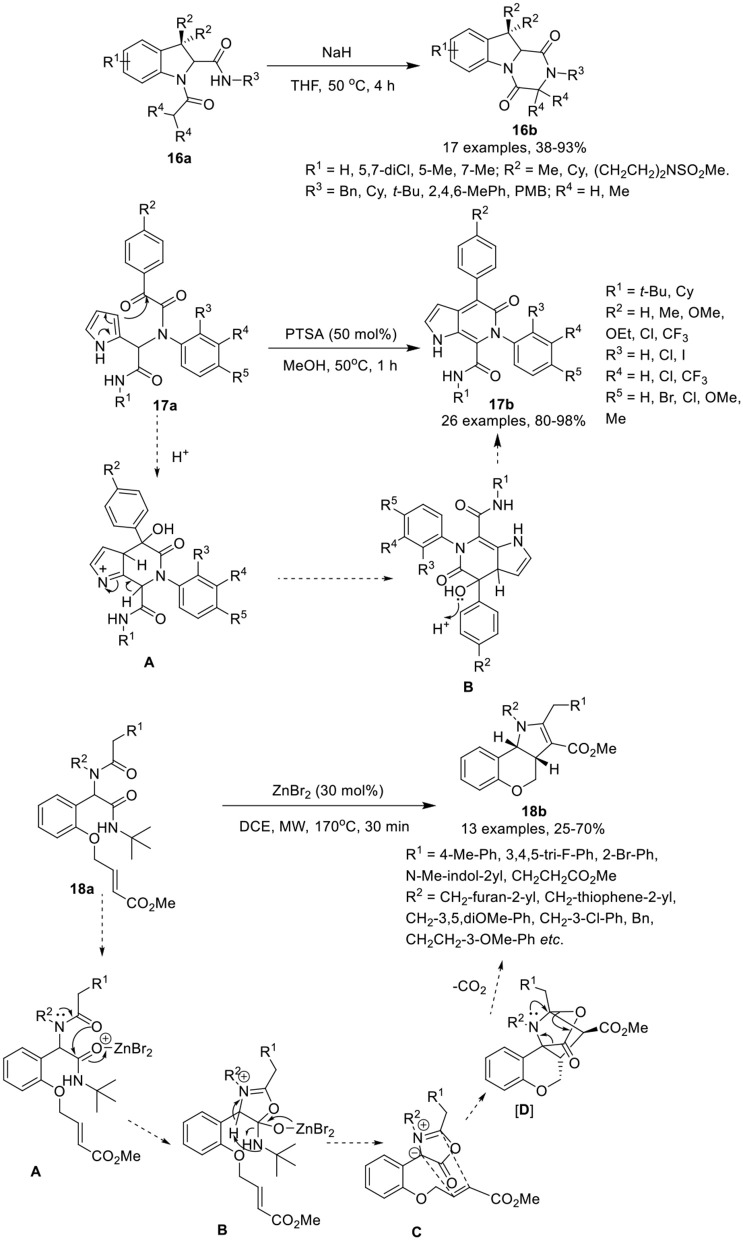
Synthesis of substituted tetrahydropyrazino[1,2-*a*]indole-1,4-diones **16b**, pyrrolo[2,3-c]pyridines **17b** and tricyclic chromenopyrroles **18b**.

Krasavin et al. (Golubev and Krasavin, 2017) have developed an efficient synthetic protocol to access sterically encumbered tricyclic peptidomimetics **16b** *via* intramolecular nucleophilic substitution reaction of Joullie-Ugi-adduct **16a** in the presence of NaH in THF at 50°C in good to excellent yields (Scheme 8). Under the optimized conditions, bulky substituents on the amide nitrogen such as *tert*-butyl or mesityl, did not affect the reaction and provided good product yields. The remaining substituents on the Joullie-Ugi-adducts **16a** did not affect the reaction output significantly. These tricyclic peptidomimetics may be useful as small molecule-ligands for peptidergic biological targets, including G-protein coupled receptors.

VenkataPrasad et al. (VenkataPrasad et al., 2017) have developed an efficient and facile post-Ugi condensation approach to access the pyrrolo[2,3-*c*]pyridines **17b** in excellent yields, using 50 mol% PTSA in methanol at 50°C (Scheme 8). Under the optimized conditions, diversely substituted Ugi-adducts **17a** provided the desired products, in moderate to excellent yields. Use of Ugi-adducts bearing CF_3_ on the aniline or on the phenyl glyoxylic acid, provided the respective pyrrolo[2,3-*c*]pyridones in excellent yields. However, use of NO_2_-substituted aniline did not provided the Ugi-adduct, whereas Ugi-adducts derived from phenylglyoxalic acid bearing a NO_2_-group failed to cyclize under the optimized conditions. The reaction proceeds *via* protonation of α-keto group of the Ugi-adduct, followed by the electrophilic addition on the pyrrole ring to give intermediate [**A**], which subsequently underwent dehydration to afford the desired compound *via* intermediate [**B**]. This approach has many advantages including practical simplicity, high atom economy and short reaction time.

Sieburth et al. (Srinivasulu et al., 2018) have developed a serendipitous one-pot protocol for the diastereoselective construction of tricyclic chromenopyrroles **18b** from Ugi-adducts **18a** in moderate to good yields, using ZnBr_2_ as catalyst in DCE under MW irradiation (Scheme 8). Under the optimized conditions, Ugi-adducts **18a** derived from variously substituted methylamines and phenylacetic acids were found well tolerable and afforded the desired products in moderate to good yields. However, the use of benzylamine derived Ugi-adducts provided low product yields. Substitutions on the nitrogen of the Ugi-adduct have little effect on the product output and provided good product yields. Ugi-adducts derived from 2-indole carboxylic acid also gave moderate product yields. Additionally, the Ugi-adducts prepared from *D*- and *L*-phenylalanine and *L*-3,4-dimethoxyphenylalanine underwent the reaction smoothly and furnished the corresponding products as a mixture of diastereomers in moderate yields, in spite of steric hindrance of the amine. The reaction proceeds through the coordination of ZnBr_2_ that catalyzes the *O*-acylation by the tertiary amide [**A**], followed by rearrangement of the resultant tetrahedral intermediate [**B**]. Abstraction of the benzylic proton led to the formation of azomethine ylide [**C**]. Subsequent intramolecular [3 + 2]-cyclization delivered the highly strained intermediate [**D**], which undergoes expulsion of carbon dioxide and 1,4-proton migration to give the desired chromenopyrrole **18b**.

### Five-membered heterocycles fused with medium-sized heterocyclic systems

**Scheme 9:**
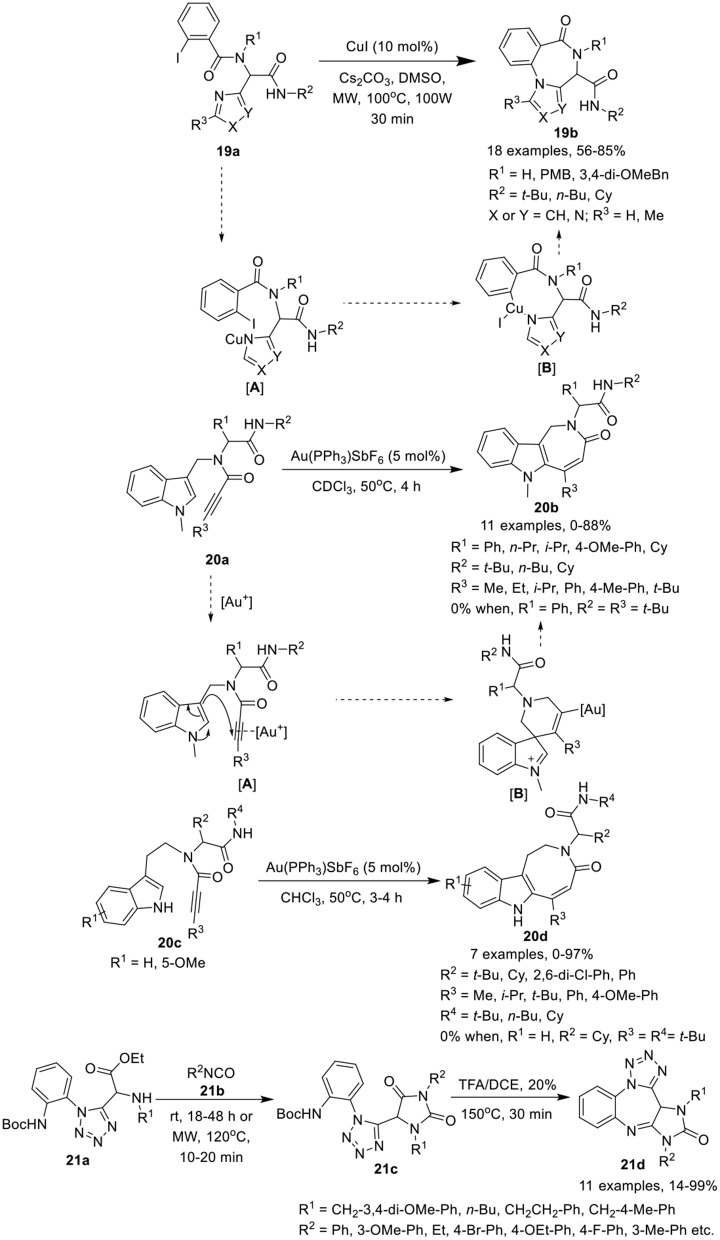
Synthesis of 4*H*-benzo[*f* ]imidazo[1,4]diazepin-6-ones **19b**, indoloazepinones **20b** and indoloazocinones **20d**, and imidazo-tetrazolodiazepinones **21d**.

Van der Eycken et al. (Li et al., 2014b) have developed an efficient copper-catalyzed post-Ugi intramolecular Ullmann-coupling strategy to give access to 4*H*-benzo[*f* ]imidazo[1,4]diazepin-6-ones **19b** in moderate to good yields using Cs_2_CO_3_ as a base in DMSO at 100°C under MW irradiation (Scheme 9). Under the optimized conditions, Ugi-adducts **19a** derived from diversely substituted aromatic acids and amines were found suitable for this reaction and gave access to the corresponding products in moderate to high yields. Use of Ugi-adducts obtained from C-2 or C-5 substituted imidazole-4-carbaldehyde did not underwent the reaction and resulted in the decomposition of the starting Ugi-adduct, whereas Ugi-adducts assembled from imidazole-2-carbaldehyde furnished the corresponding products in moderate to high yields. The reaction takes place through the formation of intermediate [**A**] *via* coordination of copper(I) iodine with the amine of the Ugi-adduct **19a**, which subsequently inserts into the aryl iodine bond, followed by reductive elimination of the resultant intermediate [**B**] generating 4*H*-benzo[*f* ]imidazo[1,4]diazepin-6-one **19b**.

Modha et al. (Vachhani et al., 2015) have developed a post-Ugi regioselective intramolecular carbocyclization approach to afford diversely substituted indoloazepinones **20b** and indoloazocinones **20d** in good to excellent yields, using Au(PPh_3_)SbF_6_ as catalyst in CDCl_3_ at 50°C (Scheme 9). Under the optimized conditions, *endo*-dig cyclization afforded the indoloazepinones **20b** in good to excellent yields. Ugi-adducts **20a** derived from aliphatic or aromatic aldehydes were well tolerated. Similarly, bulky substituents on the alkyne such as ethyl, isopropyl, and aryl underwent the reaction smoothly. However, the Ugi-adduct derived from a *tert*-butyl substituted alkyne, failed to afford the corresponding cyclized product. Interestingly, the Ugi-adduct derived from a terminal alkyne, provided the 6-*exo*-dig product. Additionally, intramolecular hydroarylation of Ugi-adducts **20c** under the optimized conditions afforded the indoloazocinones **20d** in good yields except the *tert*-butyl substituted alkyne. The regioselective intramolecular carbocyclization reaction was believed to progress through π-coordination of the cationic gold with the alkyne [**A**], followed by nucleophilic attack by the 3-position of the indole on the π-activated internal alkyne, in an *endo*-dig fashion, in contrast to the terminal alkyne (*exo*-dig fashion). Intermediate [**B**] was formed, which subsequently underwent a 1,2-shift and after deprotonation and protodeauration led to the formation of the indoloazepinone.

Hulme et al. (Medda et al., 2015) have developed a facile and concise route for post-condensation modifications of Ugi-azide adducts **21a** to give the imidazotetrazolodiazepinones **21d** in modest to excellent yields. The reaction proceeds by treatment of tetrazole **21a** with an excess of isocyanate **21b** in ethanol at rt, followed by ring closure of the resultant hydantoin **21c** with TFA in DCE under MW irradiation at 150°C, furnishing the desired imidazotetrazolodiazepinones **21d** (Scheme 9). Under the optimized conditions, diversely substituted Ugi-azide adducts **21a** afforded the hydantoin products **21c** in modest to high yields. Adducts derived from isocyanates bearing a 3-methoxyphenyl or an ethyl substituent yielded the desired hydantoin under MW irradiation at a slightly lower temperature of 120°C. Ugi-adducts bearing aliphatic substituents were well tolerated, affording the desired products in higher yields compared to adducts with aromatic substituents. Ring closure and acid-mediated Boc-removal proceeded smoothly under MW irradiation to afford the corresponding products in modest to excellent yields.

**Scheme 10:**
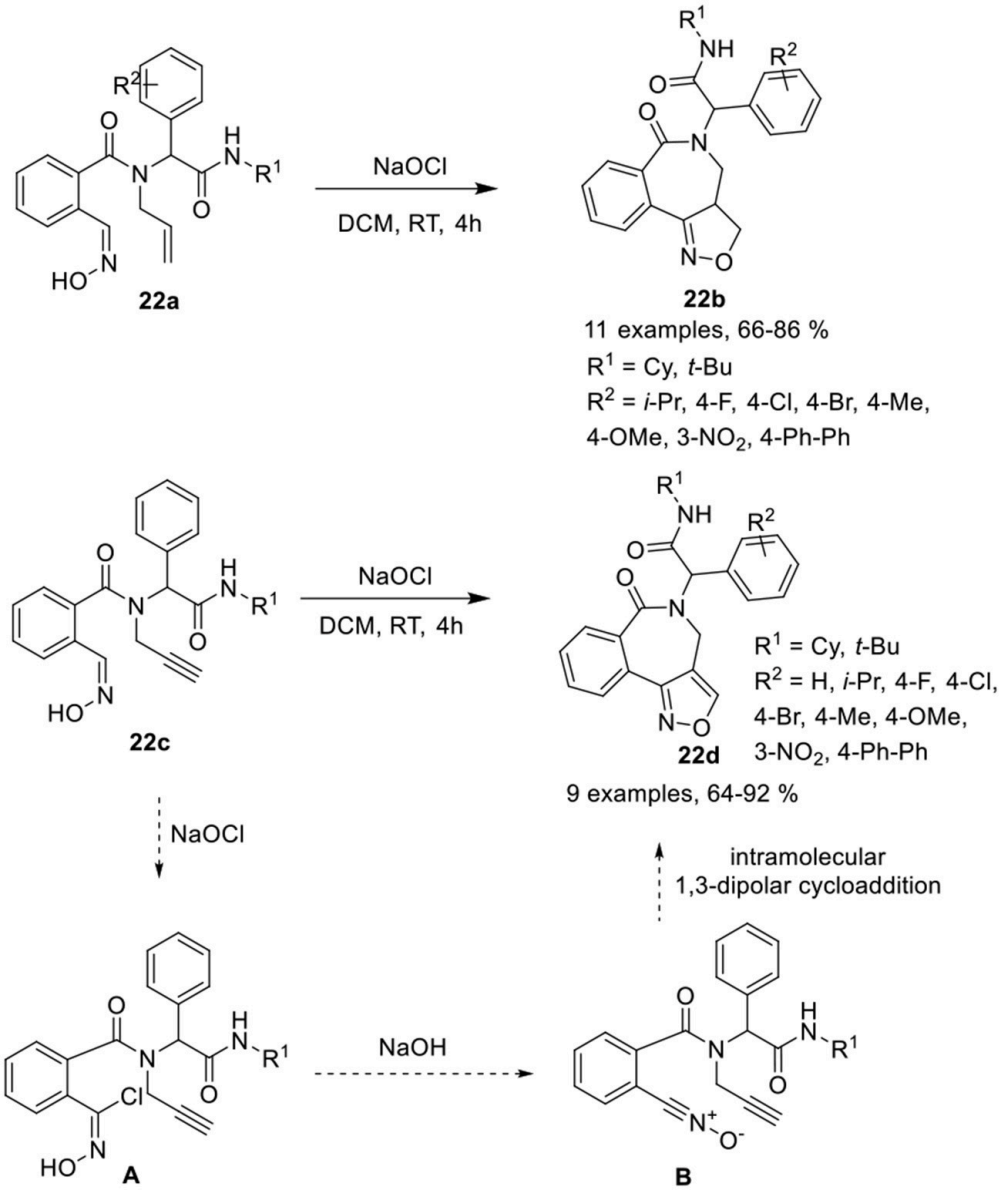
Synthesis of isoxazolino-benzazepines **22b** and isoxazolo-benzazepines **22d**.

Balalaie et al. (Balalaie et al., 2017b) have reported a diversity-oriented access to isoxazolino-benzazepines **22b** and isoxazolo-benzazepines **22d** in good to excellent yields and with high diastereoselectivities (≈95) *via* post-Ugi heteroannulation reaction involving intramolecular 1,3-dipolar cycloaddition of 2-((hydroxyimino)methyl)benzoic acid **22a** and **22c**, respectively. The nitrile oxide reacted with alkenes or alkynes in the presence of sodium hypochlorite (NaOCl) in DCM at rt (Scheme 10). Under the optimized conditions, Ugi-adducts derived from aldehydes bearing electron-donating or electron-withdrawing groups provided isoxazolino- and isoxazolo-benzazepines in good to high yields and with high diastereoselectivities. The use of aliphatic aldehydes in both reactions provided low product yields. Ugi-adducts derived from bulky *tert*-butyl isonitriles provided reduced product yields compared to cyclohexyl isonitrile derivatives. The reaction takes place through the chlorination of the oxime group in the Ugi-adduct to chloroxime [**A**], followed by removal of the proton from OH by *in situ*-generated NaOH, and loss of the chloride group resulting in the formation of nitrile oxide [**B**]. Subsequent intramolecular 1,3-dipolar cycloaddition of nitrile oxide with the alkene or alkyne group afforded the corresponding product.

### Six-membered heterocycles fused with six-membered carbocyclic systems

**Scheme 11:**
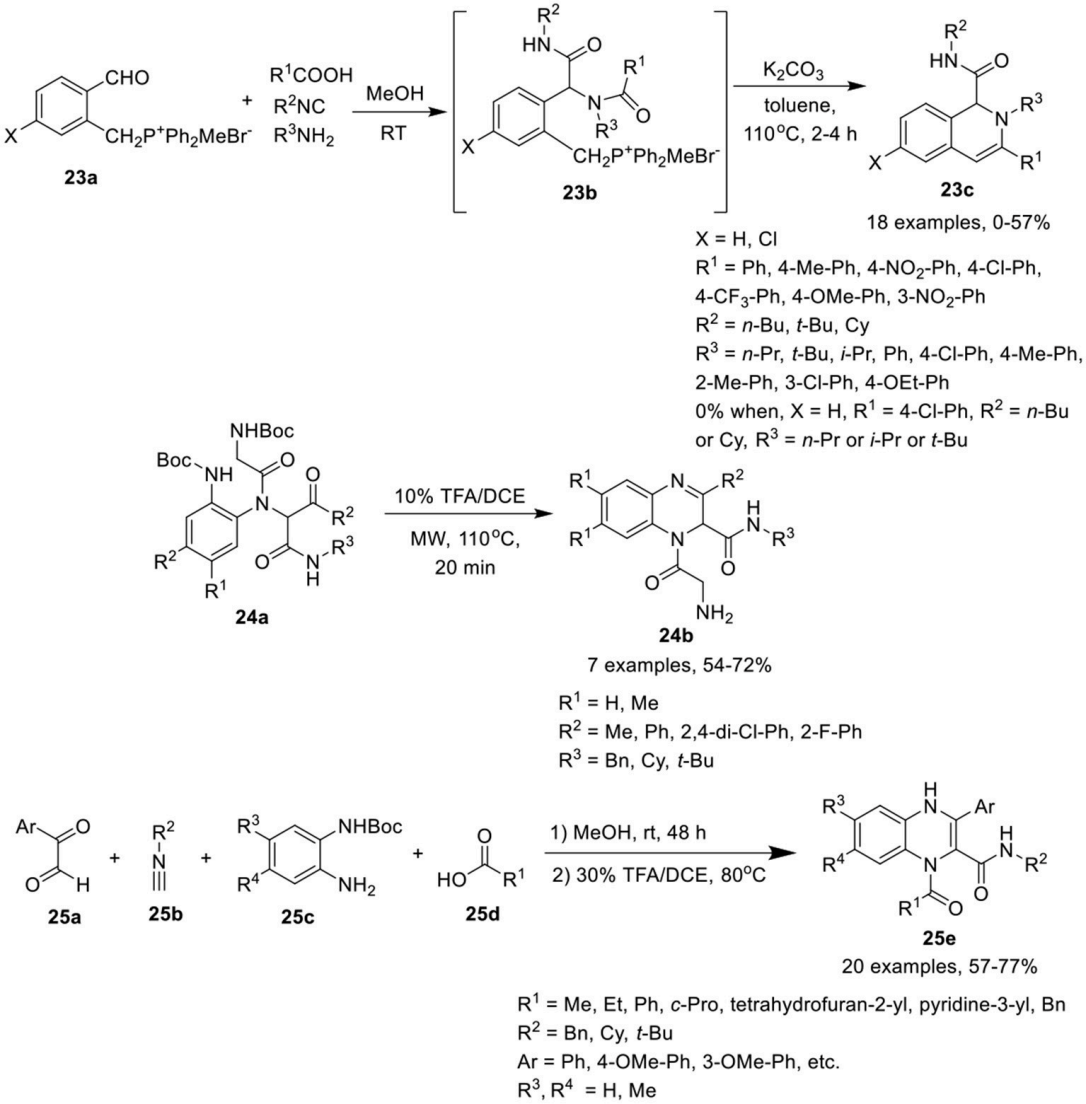
Synthesis of 1,2-dihydroisoquinolines **23c**, quinoxalines **24b** and quinoxalines **25e**.

Ding et al. (Wang et al., 2016) have developed a sequential Ugi condensation/Wittig reaction of phosphonium salt **23a** to give access to 1,2-dihydroisoquinolines **23c** in moderate yields in a one-pot fashion. Ugi-adducts **23b** prepared from phosphonium salt **23a** underwent intramolecular Wittig reaction in the presence of K_2_CO_3_ in toluene under reflux conditions (Scheme 11). The reaction proceeded smoothly when formic acid or aromatic acids were utilized as acid components. Bulky substituent derived from *t*-butyl isonitrile and aromatic amines were well tolerated under the optimized conditions. However, adducts derived from aliphatic amines failed to afford the desired products. The substituents on the phosphonium salt **23b** did not influence the product yield.

Chen et al. (Li et al., 2017) have utilized a facile Ugi/deprotection/cyclization (UDC) strategy, followed by a nucleophilic aromatic substitution reaction to give access to diverse quinoxalines **24b** in moderate to good yields under MW irradiation (Scheme 11). The reaction proceeded smoothly using diversely substituted Ugi-adducts, providing good yields by deprotection and cyclization of Ugi-adducts **24a** using TFA in DCE under MW irradiation at 110°C. Ugi-adducts derived from differently substituted diamines, isonitriles and aromatic aldehydes were found to be well tolerated under the optimized conditions.

Sotelo et al. (Azuaje et al., 2014) have developed a concise one-pot Ugi-based approach to access the quinoxalines **25e** in excellent yields by Ugi-4CR of glyoxals **25a**, isocyanides **25b**, mono-Boc protected phenylenediamines **25c**, and acids **25d** in methanol at rt, followed by Boc-removal using 30% TFA/DCE at 80°C, and subsequent cyclization (Scheme 11). A variety of substituents on all the four building blocks for the Ugi reaction were well tolerated. Bulky substituents on carboxylic acids or isocyanides afforded high yields. However, substitution on the mono-Boc protected phenylenediamines slightly reduced the yield. Further, aromatic acids were better tolerated than aliphatic acids.

### Seven-membered heterocycles fused with six-membered carbocyclic systems

**Scheme 12:**
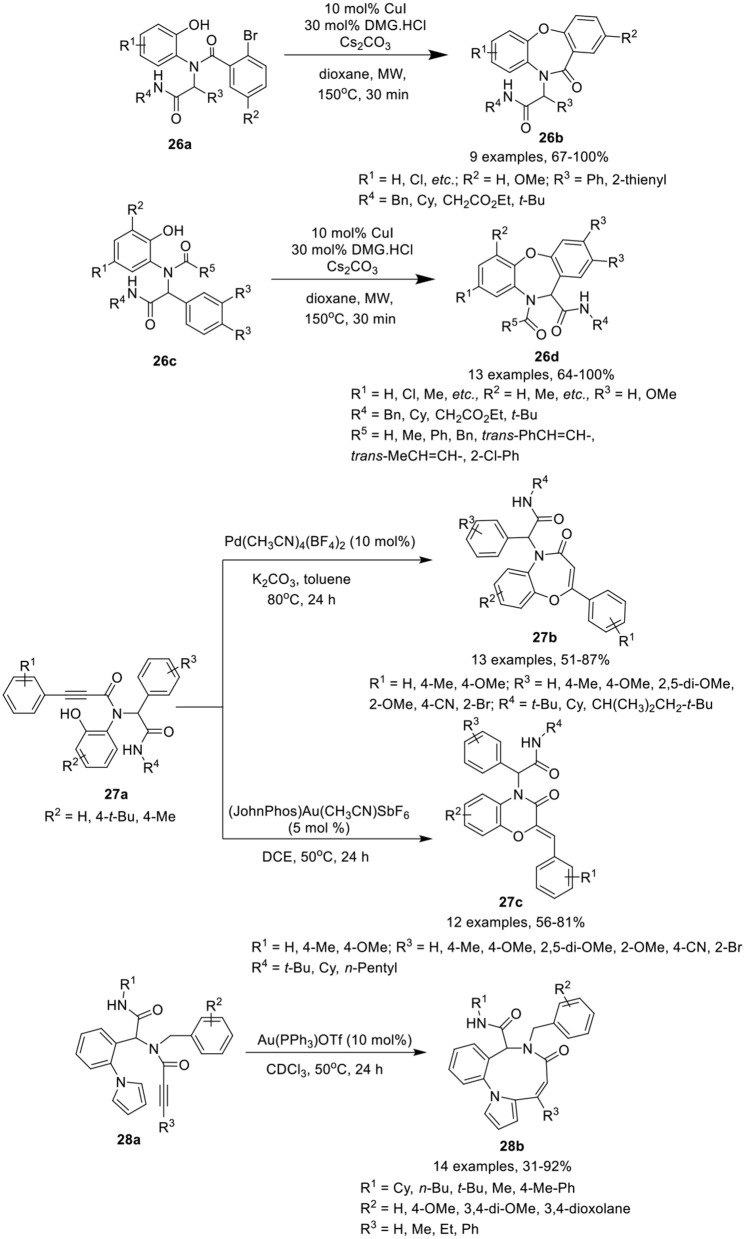
Synthesis of dibenz[*b*,*f* ][1,4]oxazepin-11(10*H*)once **26b** and dibenze[b,f][1,4]oxazepin-11(10*H*)-carboxamides **26d**, benzoxazepinones **27b** and benzoxazinones **27c**, and benzo[*b*]pyrrole[2-1-*i*][1,5]diazonin-7(6*H*)-once **28b**.

Dai et al. (Shi et al., 2016) have developed an efficient MW-assisted intramolecular Ullmann etherification of Ugi-adducts **26a** and **26c** using CuI alone or in combination with *N*,*N*-dimethylglycine HCl (DMG·HCl) as catalyst, in the presence of Cs_2_CO_3_ in dioxane, to give access dibenz[*b,f* ][1,4]oxazepin-11(10*H*)ones **26b** and dibenz[*b,f* ][1,4]oxazepin-11(10*H*)-carboxamides **26d** in good to excellent yields and with excellent chemoselectivity (Scheme 12). Under the optimized conditions, most of the reactions proceed smoothly in the absence of ligand under MW irradiation, whereas addition of DMG.HCl as a ligand improves the product yields significantly. Substrates derived from bulky anilines such as 2-naphthylamine, were well tolerated and afforded high product yields. Importantly, for thienyl containing Ugi-adducts, addition of DMG.HCl as ligand was found essential to access the corresponding product. The reaction exhibited excellent chemoselectivity under MW irradiation at elevated temperature. Ugi substrate derived from variety of amines, isocyanides, aldehydes and acids were found well tolerated, providing the products in excellent yields. Interestingly, under these reaction conditions, preference for intramolecular Ullmann etherification was observed over intramolecular Goldberg amidation.

Sharma et al. (Singh et al., 2018) have reported a catalyst-controlled selective intramolecular 7-*endo*-dig and 6-*exo*-dig post-Ugi cyclization of Ugi-adducts **27a** to afford the benzoxazepinones **27b** and benzoxazinones **27c**, respectively, with high regioselectivity. The cyclization of Ugi-product **27a** was performed using Pd(CH_3_CN)_4_(BF_4_)_2_ as catalyst in toluene facilitated the formation of benzoxazepinones **27b** in high yields with exclusive 7-*endo*-dig selectivity. However, switching the catalyst to 5 mol% of Echavarren's gold(I) catalyst [JohnPhosAu-(CH_3_CN)]SbF_6_ in DCE resulted in the preferential formation of benzoxazinones **27c** in high yields with 7-*exo*-dig selectivity (Scheme 12). Under the optimized conditions for the formation of benzoxazepinones and benzoxazinones, Ugi-adducts derived from various alkynes, isonitriles, aldehydes, and amines underwent the reaction smoothly with high selectivity. Several adducts derived from aldehydes bearing electron-donating or electron-withdrawing substituents underwent the reaction smoothly and yielded the corresponding products in moderate to excellent yields. However, adducts from aldehydes bearing an electron-withdrawing substituent such as cyano, bromo, and bis-amide afforded relatively lower yields for 7-*endo* dig cyclization. No specific electronic effect was observed for the cyclization of Ugi-adducts derived from different isocyanides and 2-aminophenol and afforded the corresponding benzoxazepinones and benzoxazinones in good to high yields. However, Ugi-adducts derived from phenyl substituted alkynes bearing electron-donating groups afforded the corresponding products in slightly lower yields.

### Nine-membered heterocycles fused with five-membered heterocycles

Van der Eycken et al. (Li et al., 2014a) have reported an efficient gold-catalyzed intramolecular hydroarylation reaction of Ugi-adduct **28a** for the regioselective construction of the fused nine-membered ring in benzo[*b*]pyrrolo[2,1-*i*][1,5]diazonin-7(6*H*)-ones **28b** in good to excellent yields using 10 mol% Au(PPh_3_)OTf in CDCl_3_ at 50°C (Scheme 12). Under the optimized reaction conditions, Ugi-adducts derived from diversely substituted alkynes, isocyanides, aldehydes, and amines were found compatible and underwent the intramolecular hydroarylation reaction smoothly, providing access to the corresponding benzo[*b*]pyrrolo[2,1-*i*][1,5]diazonin-7(6*H*)-ones **28b** in good yields. Ugi-adducts from bulky phenyl-substituted alkynes or terminal alkynes afforded moderate product yields. Cyclization of the Ugi-adduct bearing an indole moiety (from aldehyde component) resulted in decomposition of the Ugi-adduct.

### Tricyclic fused heterocycles

**Scheme 13:**
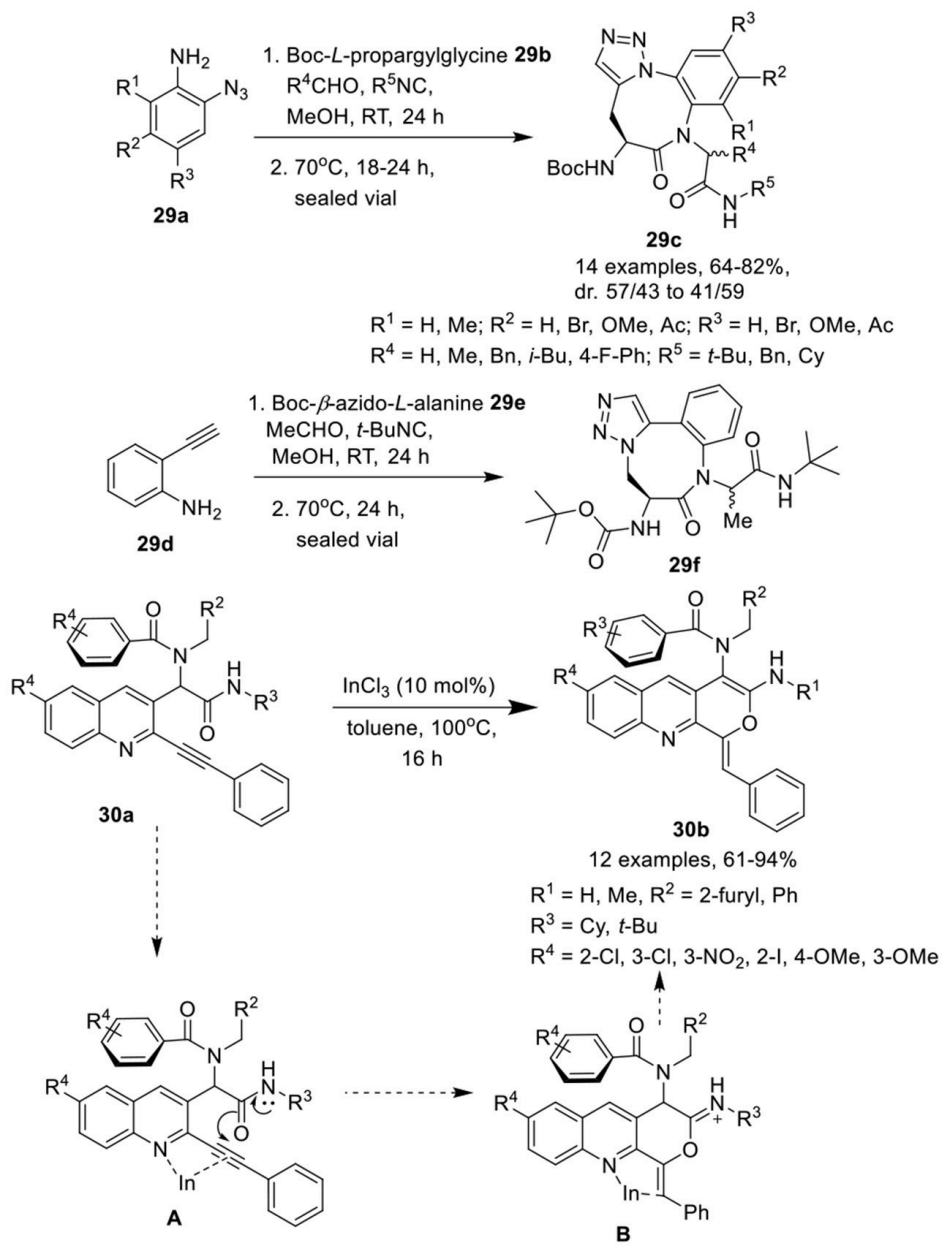
Synthesis of amino-benzotriazocine-bearing dipeptides **29c, 29f** and tricyclic pyranoquinolines **30b**.

Jida et al. (Barlow et al., 2016) have developed an efficient, diversity-oriented, one-pot approach to access amino-benzotriazolodiazocine-bearing dipeptides **29c** in good to high yields and with good diastereoselectivity. This catalyst-free reaction of an azidoaniline **29a**, an isocyanide, an aldehyde and a *Boc*-propargylglycine **29b**, proceeded well in methanol at rt and was followed by a thermal azide-alkyne Huisgen cycloaddition reaction at 70°C (Scheme 13). Variedly substituted azidoanilines were well tolerated and afforded the corresponding products in good yields without any influence on the diastereoselectivity. Aldehydes were preferred over ketones under the optimum conditions for Ugi reaction. Among differently substituted isocyanides, *t*-Bu-substituted isocyanides yielded the corresponding products in 24 h. However, benzyl- and cyclohexyl-substituted isocyanides required extended reaction times for completion of the reaction. An isomer **29f** with different triazole orientation was obtained in high yield when Boc-β-azido-*L*-alanine **29e** and commercially available ethynyl aniline **29d** were used in place of the functionalized azidoanilines **29a** and Boc-*L-*propargylamine **29b**.

Balalaie et al. (Balalaie et al., 2017c) have reported an expedient synthesis of pyranoquinolines **30b** *via* InCl_3_-catalyzed post-Ugi intramolecular hydroamidation of alkyne containing Ugi-adduct **30a** in toluene at 100°C (Scheme 13). The reaction proceeded smoothly under the optimized conditions and accommodated a wide range of Ugi-adducts irrespective of the positional and electronic influence of the substituents, providing the pyranoquinolines in good to excellent yields. The reaction proceeded *via* π-activation of the triple bond using InCl_3_ and delivered intermediate **A**. The 6-*exo*-dig that led to the pyran ring was preferred over the 7-*endo*-dig cyclization (that should result in the oxepine ring). Nucleophilic addition of the amide oxygen to the internal carbon of the triple bond led to the formation of the desired product through intermediate **B**.

## Spiro-polyheterocycles

**Scheme 14:**
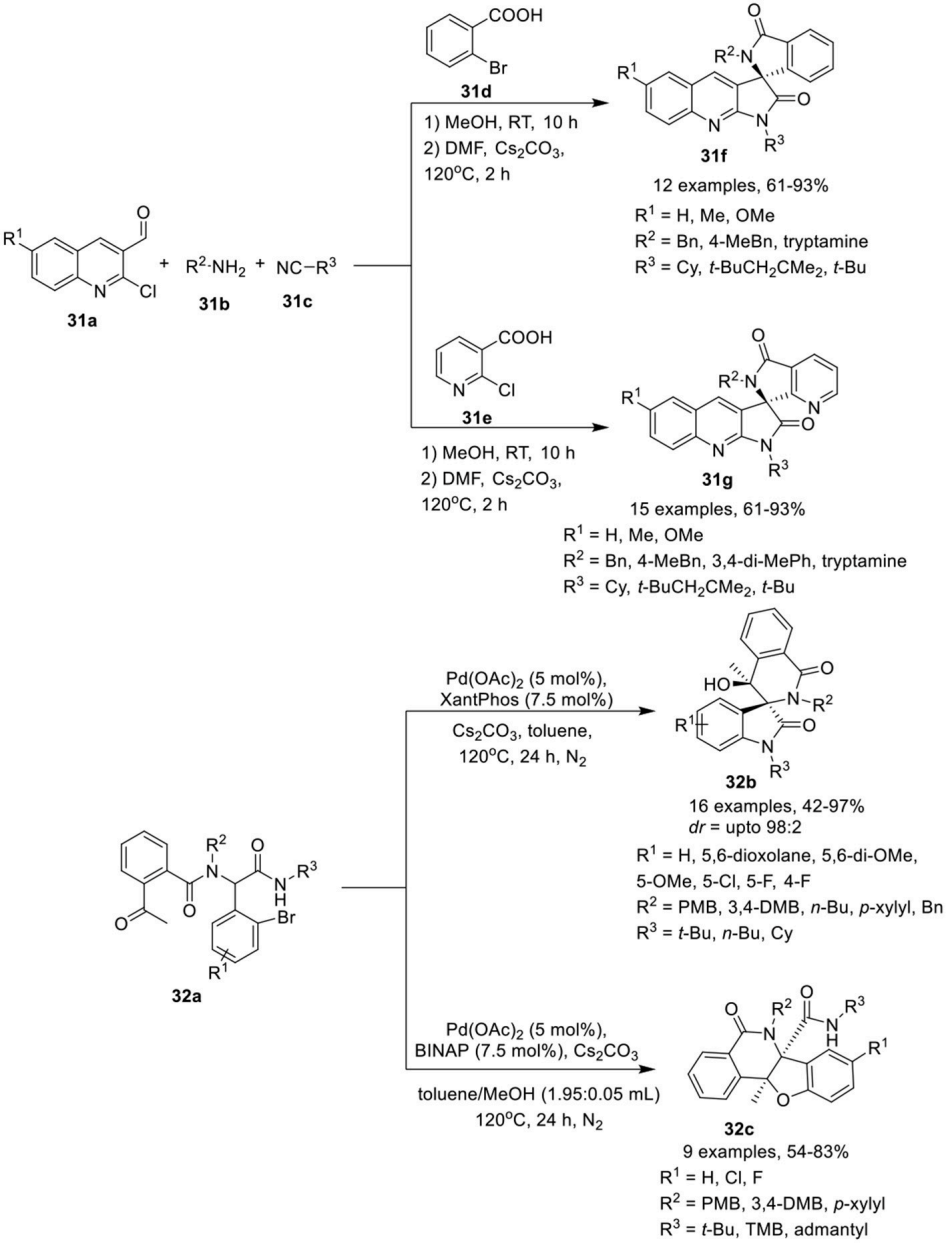
Synthesis of spiropyrroloquinoline-isolinone **31f** and their aza-azalogs **31g** and (spiro)polyheterocycles **32b** and **32c**.

Ghandi et al. (2015) have developed a one-pot Ugi metal-free intramolecular bisannulation reaction of 2-chloroquinoline-3-carbaldehydes **31a**, with amines **31b**, acids **31d**, or **31e** and isocyanide **31c** to give access to spiro[isoindoline-1,3′-pyrrolo[2,3-*b*]quinoline]-2′,3(1′*H*)-diones **31f** and their aza-analogs, spiro[pyrrolo[2,3-*b*]quinoline-3,7′-pyrrolo[3,4-*b*]pyridine]-2,5′(1*H*,6′*H*)-diones **31g**, in good to high yields, using Cs_2_CO_3_ as base in DMF at 120°C for 2 h (Scheme 14). Under the optimized conditions, adducts derived from aliphatic amines provided access to the corresponding products in high yields compared to aromatic amines. Easy cyclization in high yields was achieved for sterically hindered amides, such as 2,4,4-trimethylpentyl amide, suggested insensitive of the reaction to steric hindrance around the amide. Among the aldehydes, unsubstituted or modest electron-donating methyl substituted aldehydes resulted in higher product yields, compared to aldehydes bearing a stronger electron-donating methoxy group. The present approach is very attractive providing molecular diversity and synthetic simplicity with high atom economy.

Sharma et al. (Li et al., 2016) have reported an efficient and diversity-oriented ligand-controlled intramolecular palladium-catalyzed domino post-Ugi Buchwald–Hartwig/Aldol reaction sequence for the construction of (spiro)polyheterocycles **32b** and **32c** using Pd(OAc)_2_ as catalyst and XantPhos or BINAP as ligand under basic conditions of Cs_2_CO_3_ in toluene at 120°C (Scheme 14). A variety of Ugi-adducts **32a** derived from 2-acetyl benzoic acid, afforded the spiro[indoline-3,3′-isoquinoline]-diones **32b** in modest to excellent yields and with moderate diastereoselectivity. Diversely substituted aromatic aldehydes bearing electron-donating or electron-withdrawing groups were well tolerated. Particularly, fluorine substitution at the *o*- or *m*-position of the aldehydes yielded the corresponding products with good diastereoselectivity. A bulky substituent such as a *tert*-butyl amide on the Ugi-adduct has a significant effect on the reaction outcome, resulting in the formation of polycyclic **32c** as a side product. Interestingly, a switch of ligand from XantPhos to BINAP resulted in the regioselective synthesis of tetrahydrobenzofuro-isoquinoline **32c** in high yields, using a mixture of toluene and methanol (1.95: 0.05 mL) at 120°C. Interestingly, regioselective cyclization for this reaction was only observed with a bulky secondary amide group bearing a *tert*-butyl, a 1,1,3,3-tetramethyl butyl or an adamantyl group. However, linear and cyclic amides led to the formation of a mixture of spirocyclic and polycyclic products.

**Scheme 15:**
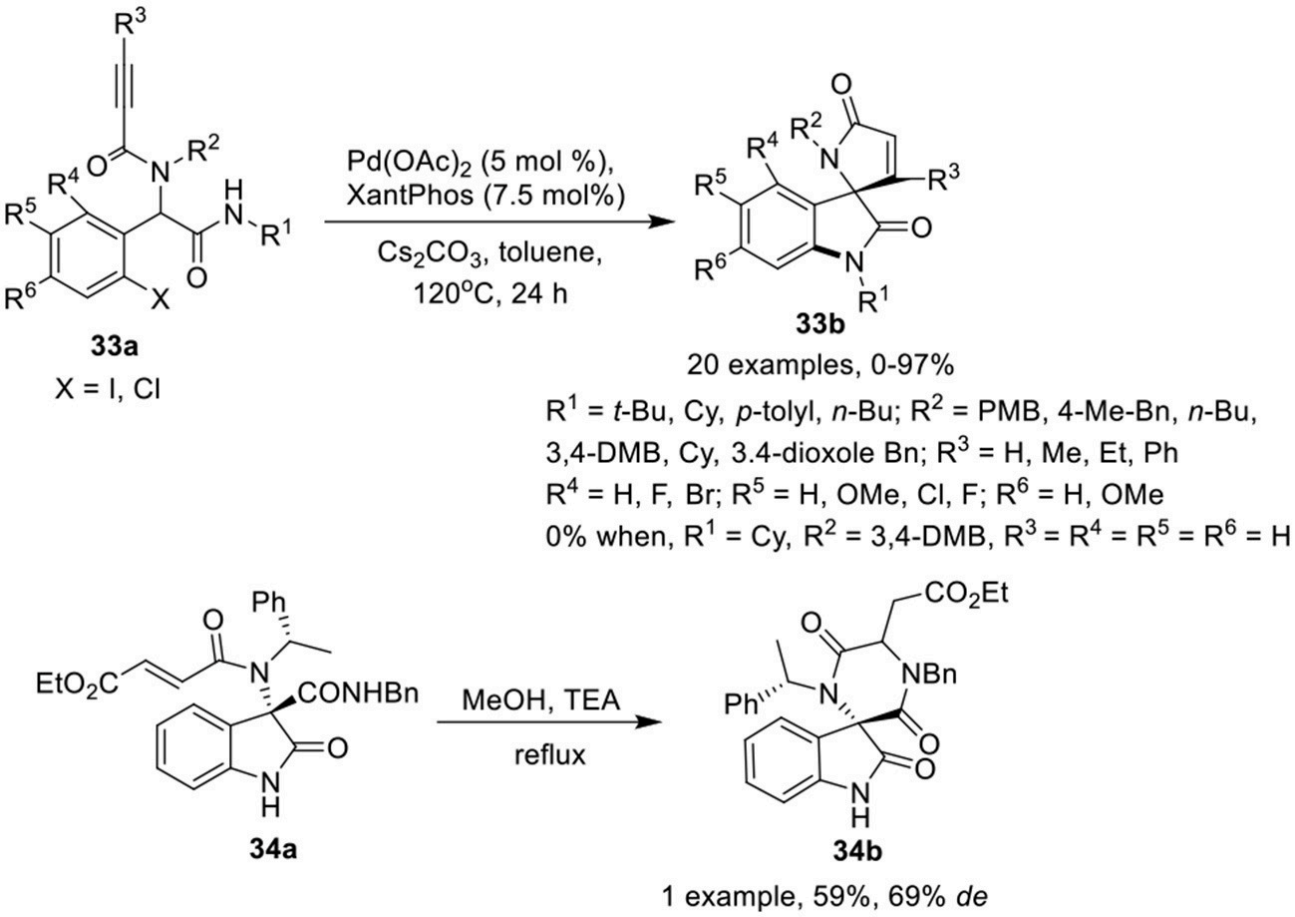
Synthesis of spiro[indoline-3,2′-pyrrole]-2,5′-diones **33b** and spiro-diketopiperazines **34b**.

Sharma et al. (2014) have reported a facile post-Ugi domino Buchwald–Hartwig/Michael reaction of Ugi-adduct **33a** to give access to functionalized spiro[indoline-3,2′-pyrrole]-2,5′-diones **33b** in low to excellent yields using Pd(OAc)_2_ (5 mol %) as catalyst, Xantphos (7.5 mol %) as ligand, and Cs_2_CO_3_ as base in toluene at 120 °C (Scheme 15). Halogen substituted aromatic aldehydes have a significant effect on the reaction outcome as the Ugi-adduct derived from *o*-iodo substituted benzaldehyde afforded the product in higher yields than its chloro substituted counterpart. Electron-donating or electron-withdrawing substituents at the *o, m*, or *p*-position of the aryl ring of the aldehydes were well tolerated. The reactions proceeded smoothly with Ugi-adducts obtained from aliphatic isonitriles. However, the use of aromatic isonitriles provided low product yields. Interestingly, Ugi-adducts obtained from propiolic acid did not provided the desired spiro-product. Additionally, use of α,β-unsaturated acids (instead of 2-alkynoic acids) resulted in spirooxindoles in good yields with adequate diastereoselectivities. Ugi-adducts derived from differently substituted amines were well tolerated.

Silvani et al. (Lesma et al., 2014) have developed an intramolecular aza-Michael reaction for the post-Ugi cyclization of chiral 3,3-disubstituted 3-aminooxindole **34a** to access the spiro-diketopiperazine **34b** in moderate yield and good diastereoselectivity (Scheme 15). The spiro-diketopiperazine scaffold has received great recognition as pharmacologically active peptidomimetic.

## Conclusion

In conclusion, we have demonstrated the wide-spread application of the Ugi reaction for the synthesis of heterocyclic compounds, where careful selection of the starting building blocks provided the appropriate functionality for post-Ugi modifications. We have discussed various recent reports where a variety of heterocyclic systems were successfully synthesized starting from simple four, five or six-membered heterocycles to fused heterocycles and spirocyclized complex molecules. During the post-Ugi transformation, we have witnessed the application of various metallic salts of palladium, gold, indium, copper, zinc, scandium, iron and aluminum, leading to the successful transformation of appropriately substituted Ugi-adducts to heterocyclic systems. The use of modern medicinal chemistry tools such as microwave irradiation has also been successfully applied in post-Ugi transformations. Interestingly, the use of chiral ligands such as BINAP, XantPhos, and triphenylphosphine has provided stereoselectivity and chemoselectivity in such transformations. We anticipate that this review would provide in-depth understanding of the chemistry and applications of post-Ugi transformations for the synthesis of variety of heterocyclic systems. New heterocyclic systems with interesting biological activity are expected from the post-Ugi transformation in the near future.

## Author contributions

JB and RK collected the publications related to this review article, wrote the first draft of the manuscript and made subsequent corrections. EV and LV completed critical literature analysis and checked subsequent manuscript drafts.

### Conflict of interest statement

The authors declare that the research was conducted in the absence of any commercial or financial relationships that could be construed as a potential conflict of interest.
